# Resveratrol Reduces the Incidence of Portal Vein System Thrombosis after Splenectomy in a Rat Fibrosis Model

**DOI:** 10.1155/2016/7453849

**Published:** 2016-06-28

**Authors:** Meng Xu, Wanli Xue, Zhenhua Ma, Jigang Bai, Shengli Wu

**Affiliations:** ^1^Department of Hepatobiliary Surgery, the First Affiliated Hospital of Xi'an Jiaotong University, Xi'an 710061, China; ^2^Department of Public Health, Xi'an Jiaotong University School of Medicine, Xi'an 710061, China

## Abstract

*Purpose*. To investigate the preventive effect of resveratrol (RES) on the formation of portal vein system thrombosis (PVST) in a rat fibrosis model.* Methods*. A total of 64 male SD rats, weighing 200–300 g, were divided into five groups: Sham operation, Splenectomy I, Splenectomy II, RES, and low molecular weight heparin (LMWH), with the former two groups as nonfibrosis controls. Blood samples were subjected to biochemical assays. Platelet apoptosis was measured by flow cytometry. All rats were euthanized for PVST detection one week after operation.* Results*. No PVST occurred in nonfibrosis controls. Compared to Splenectomy II, the incidences of PVST in RES and LMWH groups were significantly decreased (both *p* < 0.05). Two rats in LMWH group died before euthanasia due to intra-abdominal hemorrhage. In RES group, significant decreases in platelet aggregation, platelet radical oxygen species (ROS) production, and increase in platelet nitric oxide (NO) synthesis and platelet apoptosis were observed when compared with Splenectomy II (all *p* < 0.001), while in LMWH group only significant decrease in platelet aggregation was observed.* Conclusion*. Prophylactic application of RES could safely reduce the incidence of PVST after splenectomy in cirrhotic rat. Regulation of platelet function and induction of platelet apoptosis might be the underlying mechanisms.

## 1. Introduction

Hepatic cirrhosis was most commonly caused by viral hepatitis and alcohol abuse [1] with hepatic insufficiency and portal hypertension being the most serious consequences [2]. If untreated, the 5-year survival of decompensated cirrhosis was only 15% [3]. The ideal treatment option for cirrhotic patients was liver transplantation. However, due to severe shortage of donor livers, splenectomy and pericardial devascularization were still the primary method for the patients with cirrhosis and portal hypertension to alleviate thrombocytopenia and reduce the risk of gastrointestinal hemorrhage in some countries such as China.

Portal vein system thrombosis (PVST) referred to thrombosis in the portal vein, splenic, and superior mesenteric veins or intrahepatic portal vein branches [4]. It was widely recognized as a potential fatal complication after splenectomy, leading to bowel infarction, upper gastrointestinal bleeding, and even hepatic coma [5, 6]. With the development of advanced imaging devices, it has been found that PVST after splenectomy is not a rare complication with an incidence of 5%–25% [7]. Previous studies of PVST mainly focused on the incidence, diagnosis, and treatment of PVST after splenectomy in patients with myelodysplastic syndromes [8], hemolytic anemia [9], and splenic tumors [10]. In recent years, it has been found that PVST after splenectomy also occurred in cirrhotic patients despite the presence of endogenous coagulopathy [11]. However, the pathogenesis and characteristics of PVST in these patients remain unclear. Recently, some studies showed that both pro- and anticoagulation elements were concomitantly reduced in liver cirrhosis, thus maintaining an intricate balance of coagulation [12, 13]. Under such circumstance, a series of local and systemic changes subject to splenectomy, including hemodynamic changes of the portal venous system [13], local vascular pathological changes [6], and blood hypercoagulability [6], as well as irrational use of coagulants [14], might be breaking the balance and finally contribute to the formation of PVST.

Resveratrol (trans-3,5,4′-trihydroxystilbene, RES), a natural polyphenol, was first isolated from the roots of white hellebore in 1940 [15] and is now found to be present in various plant species such as berries, peanuts, and particularly grape skins [16]. Numerous studies have demonstrated its diverse biologic effects, such as antioxidative effect [17], anti-inflammatory activity [18], antiviral activity [19], and antiplatelet aggregation activity [20]. Previous study in animal model had demonstrated that RES exerted protective effect on rats subjected to PVST via its antioxidant and antiaggregant properties [21]. However, the effect of RES on the formation of PVST after splenectomy remains unclear.

In light of these, the present study aimed to investigate the preventive effect of RES on the formation of PVST in rats after splenectomy in the context of liver cirrhosis. The findings will provide some clues that RES can be a potential antithrombotic agent for reducing PVST formation after splenectomy for cirrhotic patients.

## 2. Materials and Methods

### 2.1. Animals

Male Sprague-Dawley (SD) rats weighing between 200 and 300 g 9 to 10 weeks old were purchased from the Laboratory Animal Center of Xi'an Jiaotong University Health Science Center (Xi'an, China). Liver fibrosis was induced by intraperitoneal injection of carbon tetrachloride (CCl_4_, Sinopharm Chemical Reagent Co. Ltd. Shanghai, China; 0.6 mL/kg of body weight) in olive oil, twice a week for six weeks, and was confirmed by liver biopsy when animals were euthanized. All animals were allowed free access to water and standard laboratory chow, except for an overnight fast before undergoing surgery. All experiments were performed in accordance with the* Guide for the Care and Use of Laboratory Animals* (NIH publication number 85-23, revised in 1996). All procedures were reviewed and approved by the Ethics Committee, Xi'an Jiaotong University Health Science Center.

### 2.2. Reagents

Resveratrol was purchased from Xi'an Sino-Herb Bio-technology Company (Xi'an, China). Dimethyl sulfoxide (DMSO) and RPMI-1640 were purchased from Wuhan Boster Biological Technology, Ltd. (Wuhan, China). The RES was dissolved and sterilized in DMSO and then diluted in RPMI-1640 to 5 mg/mL.

### 2.3. Experimental Design

We estimated that a total of 64 rats would be needed to detect a difference between groups, with a one-tailed chi-square test (*α* = 0.05 and *β* = 0.20), when the forecasting incidences of PVST in nonfibrosis controls, fibrotic rats without anticoagulation, and fibrotic rats with anticoagulation were 1% [22], 40% [23], and around 10% [24], respectively. Then the numbers in each group were determined as follows with an experimental-to-control animal ratio of 2 : 1 as previously reported [25]: two groups of age-matched normal rats served as nonfibrosis controls: Sham operation group and Splenectomy group I with 8 rats in each group. Sham operation group (Sham): a 5 cm midline abdominal incision was made to expose the spleen and laparotomy was performed with no splenectomy; Splenectomy group I (Splenectomy I): splenectomy was carried out. The other 48 rats with liver fibrosis were randomized into Splenectomy group II, RES group, and LMWH group, with 16 rats in each group, as follows: Splenectomy group II (Splenectomy II): animals underwent the same surgical procedure as Splenectomy I; RES preconditioning group (RES): animals underwent the same surgical procedure as Splenectomy group I/II and received RES (50 mg/d per nasogastric tube) for 10 consecutive days before operation; and LMWH treatment group (LMWH): animals underwent the same surgical procedure as Splenectomy group I/II and received Enoxaparin (1.5 mg/kg, subcutaneously) on postoperative days (POD) 1, 2, and 3. Rats were anesthetized with an intraperitoneal injection of ketamine (75 mg/kg; Fujian Gutian Pharmaceutical Co., Ltd, Fujian, China). All animals were euthanized with an overdose of ketamine (150 mg/kg IP) followed by exsanguinations 1 week after operation. Blood was collected from the inferior vena cava in plastic tubes containing sodium heparin (1000 units/mL) as anticoagulant at a ratio of 9 : 1 v/v. Blood samples were centrifuged at 150 ×g at room temperature for 10 min and platelet-rich plasma (PRP) was taken and immediately processed. Portal vein, superior mesenteric vein, and splenic vein were dissected and opened for the detection of PVST.

### 2.4. Platelet Aggregation Evaluation

5 mL of PRP was added with equal volume of the washing buffer (140 mM NaCl, 0.5 mM KCl, 12 mM trisodium citrate, 10 mM glucose, 12.5 mM saccharose, and pH 6) and centrifuged at 800 ×g for 15 min. The pellet was resuspended in washing buffer and washed twice. Then the platelets were suspended in Krebs solution (118 mM NaCl, 25 mM NaHCO_3_, 1.2 mM KH_2_PO_4_, 1.7 mM MgSO_4_, 5.6 mM glucose, and pH 7.4) at a count of 1 × 10^8^/mL.

Platelet aggregation was initiated by adding 10 *μ*g/mL of collagen (Wuhan Boster Biological Technology, Ltd. Wuhan, China) as agonist to 0.5 mL platelet suspension and was determined using a computerized dual channel Chronolog Aggregometer (Chrono-Log Corporation, Havertown, PA) at 37°C as previously described [26]. At the end of the experiment, platelet suspensions were immediately centrifuged at 12000 ×g for 30 s and the cell-free supernatant was snap frozen in liquid nitrogen and stored at −80°C for further analysis.

### 2.5. Platelet ROS Evaluation

For the measurement of the generation of ROS by activated platelets, washed platelets in Krebs solution (1 × 10^8^/mL) were loaded with 5 *μ*M of 2′,7′-dichlorofluorescein diacetate (DCFH-DA; Sigma, Saint-Louis, Missouri, USA) for 15 min at 37°C. Then the platelet samples were centrifuged at 800 ×g for 10 min and resuspended in 500 *μ*L of Krebs solution. The ROS-generated fluorescence was read using a Becton Dickinson flow cytometer (FACSCalibur; USA) equipped with a 488 nm wavelength argon laser, 510–540 nm band pass filter, as described [27].

### 2.6. Platelet NO Evaluation

NO release by platelets during platelet aggregation was estimated by measuring NO degradation products, nitrite plus nitrate, in platelet supernatants. Briefly, frozen samples of platelets supernatant, as described earlier, were deproteinized with 3 M ZnSO4, 30% v/v, for 15 min and then centrifuged at 12,000 g for 5 min. After discarding the pellet, the clean supernatant was added with activated cadmium beads for overnight incubation at room temperature. Then, the culture was centrifuged and the supernatant was used for NO degradation products concentration determination by using a colorimetric nonenzymatic assay kit (Oxford Biomedical Research, Oxford, MI, USA) as reported previously [28].

### 2.7. Flow Cytometry Analysis

For measuring platelet apoptosis, an Annexin-V Apoptosis Detection Kit FITC (eBioscience, San Diego, CA, USA) was used according to manufacturer's instructions.

### 2.8. Statistical Analysis

Continuous data were presented as mean ± SD and categorical data were presented as frequencies. Statistical differences were calculated by Student's *t*-test or chi-square test using SPSS 11.5 statistical software (SPSS Inc., Chicago, IL, United States). A *p* value <0.05 was considered statistically significant.

## 3. Results

### 3.1. Incidence and Distribution of PVST after Splenectomy

No PVST occurred in Sham group and Splenectomy group I. For the other three groups of rats with liver fibrosis, the incidence of PVST 1 week after operation was 43.75% (7/16) in Splenectomy group II, 6.25% (1/16) in RES group, and 7.14% (1/14) in LMWH group (two rats in this group died at POD 4 and 5, resp., due to intra-abdominal hemorrhage). Both pretreatment with RES (50 mg/d per nasogastric tube for 10 days) and a short term postoperative administration of Enoxaparin (1.5 mg/kg subcutaneously on POD 1, 2, and 3) showed a significant decrease in the incidence of PVST than in Splenectomy group II (*p* = 0.037 and *p* = 0.039, resp.).

Of the 7 cases of PVST in Splenectomy group II, there were 5 in the splenic vein and 2 in the portal and splenic veins. For the one case in RES group and one case in LMWH group, it was located in the portal vein and splenic vein, respectively. It is notable that splenic vein thrombosis occurred in 8 out of 9 cases (88.9%).

### 3.2. Inhibitory Effect of RES and Enoxaparin on Rat Platelet Aggregation

**Figure 1:**
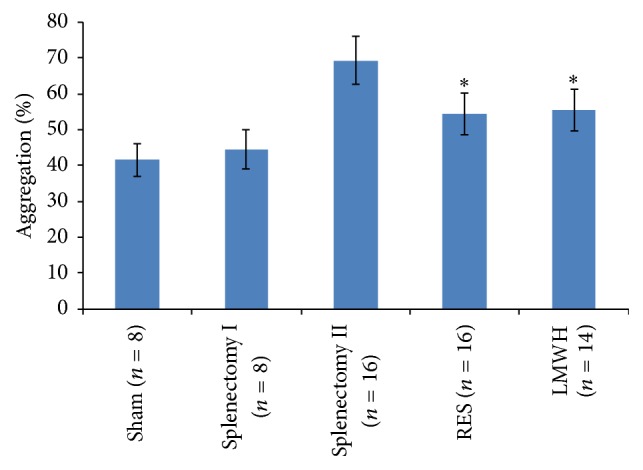
Inhibitory effects of RES and Enoxaparin on rat platelet aggregation. Rats received RES (50 mg/d per nasogastric tube) for 10 days before operation or a short term postoperative administration of Enoxaparin (1.5 mg/kg subcutaneously on POD 1, 2, and 3) and the blood was collected 1 week after operation. Washed platelets aggregation (1 × 10^8^ platelets/mL) stimulated with 10 *μ*g/mL of collagen was determined. Results are shown as mean ± SD. ^*∗*^ *p* < 0.001 compared with Splenectomy group II.

Data on the effects of RES and Enoxaparin on platelet aggregation induced by collagen are presented in Figure 1. Platelet aggregation in rats with liver fibrosis 1 week after splenectomy (Splenectomy group II) was significantly higher than that in rats without liver fibrosis (Sham and Splenectomy group I) (all *p* < 0.001). Both pretreatment with RES (50 mg/d per nasogastric tube for 10 days) and a short term postoperative administration of Enoxaparin (1.5 mg/kg subcutaneously on POD 1, 2, and 3) caused a significant reduction in collagen-induced platelet aggregation 1 week after splenectomy, compared with Splenectomy group II (all *p* < 0.001).

### 3.3. Inhibitory Effect of RES on ROS Generation in Rat Platelets

**Figure 2:**
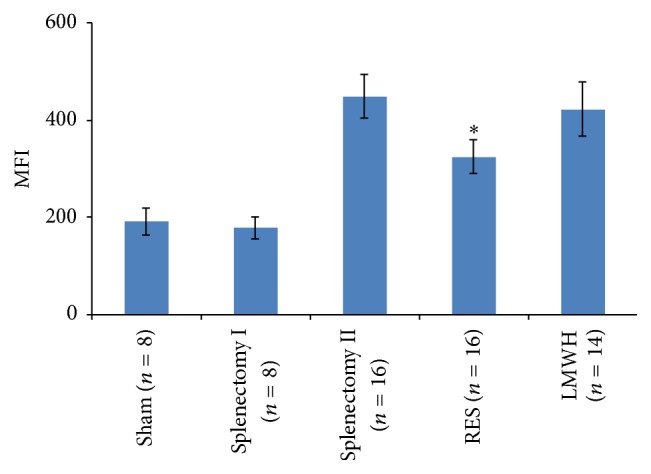
Inhibitory effect of RES on ROS generation in rat platelets. Rats received RES (50 mg/d per nasogastric tube) for 10 days before operation and ROS generation was quantified in collagen (10 *μ*g/mL)-activated platelets. Results are shown as mean ± SD. ^*∗*^ *p* < 0.001 compared with Splenectomy group II. MFI: Mean Fluorescence Index.

Compared to Sham and Splenectomy group I, ROS formation by collagen-stimulated platelets increased obviously in fibrosis rat 1 week after splenectomy (all *p* < 0.001). However, this increase in ROS formation was significantly alleviated by in vivo pretreatment with RES (*p* < 0.001), but not by postoperative administration of Enoxaparin (Figure 2).

### 3.4. Stimulating Effect of RES on NO Release in Rat Platelets

**Figure 3:**
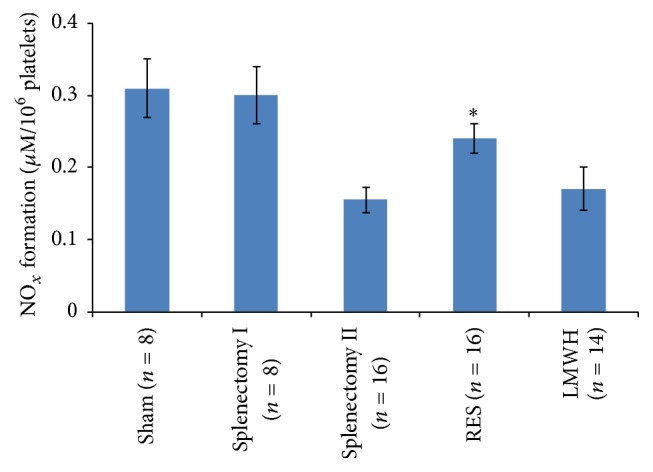
Stimulating effect of RES on NO release in rat platelets. Rats received RES (50 mg/d per nasogastric tube) for 10 days before operation and NO release by platelets during platelet aggregation was estimated in platelet supernatants. Results are shown as mean ± SD. ^*∗*^ *p* < 0.001 compared with Splenectomy group II.

Compared to Sham and Splenectomy group I, NO release during platelet aggregation decreased obviously in fibrosis rat 1 week after splenectomy (all *p* < 0.001). However, this decrease in NO release was significantly alleviated by in vivo pretreatment with RES (*p* < 0.001) but not by postoperative administration of Enoxaparin (Figure 3).

### 3.5. Induction of Rat Platelets Apoptosis by RES

**Figure 4:**
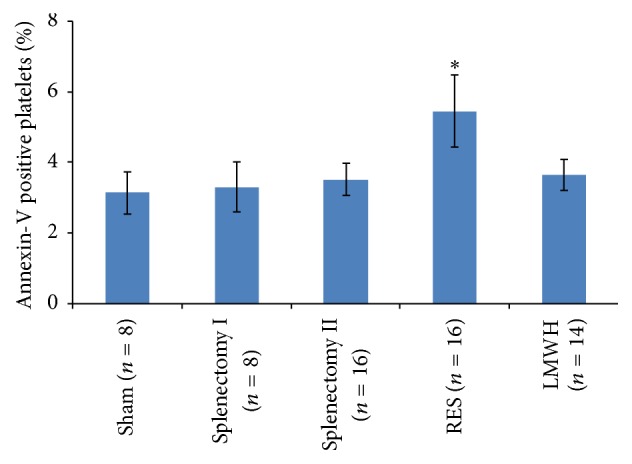
Induction of rat platelets apoptosis by RES. Rats received RES (50 mg/d per nasogastric tube) for 10 days before operation and apoptosis of rat platelets was measured by flow cytometry with an Annexin-V Apoptosis Detection Kit. Results are shown as mean ± SD. ^*∗*^ *p* < 0.001 compared with the other four groups.

As seen in Figure 4, significantly increased Annexin-V positive platelets representing phosphatidylserine (PS) exposure were observed in RES group (5.46 ± 1.02%) compared to that in Sham group (3.15 ± 0.6%), Splenectomy group I (3.31 ± 0.7%), Splenectomy group II (3.52 ± 0.45%), and LMWH group (3.66 ± 0.44%) (all *p* < 0.001), suggesting more platelet apoptosis occurring in rats pretreated with RES.

## 4. Discussion

Although the pathogenesis of PVST following splenectomy is still controversial, it is generally agreed that it is related to blood hypercoagulability [6], hemodynamic changes of the portal venous system [13], local vascular pathological changes [6], and irrational use of coagulants [14], and so forth. Lower preoperative platelet counts, postoperative thrombocytosis, wider preoperative portal/splenic vein diameter, prolonged prothrombin time (PT), periesophagogastric devascularization, and mutation of prothrombin genes and deficiency in protein C and protein S have been considered as risk factors of PVST after splenectomy [23, 29].

For cirrhotic patients, preoperative platelet count was generally lower than normal due to concurrent hypersplenism. Under such condition, removal of spleen would lead to a platelet rebound phenomenon and the soaring count and augmented aggregation competence of platelet after operation would contribute to the hypercoagulable state, which, in combination with other underlying causes, may synergistically result in the formation of PVST.

Currently, the prophylaxis of PVST after splenectomy in liver cirrhosis remains controversial. The main concern is that prophylactic anticoagulation might induce relevant complications, mainly related to bleeding. So far, the management of PVST after splenectomy is mainly based on individual experience [29]. Several recent pilot studies had demonstrated the feasibility, safety, and efficacy of prophylactic anticoagulation with decreased incidence of PVST and low rate of bleeding complications comparable to controls [30–34]. However, due to the small number of cases in individual studies, more results with adequate power are required to confirm these observations.

The activation of thrombin is a crucial step in the formation of thrombosis. LMWH could suppress the activation of thrombin via suppressing coagulation active factor X. Moreover, LMWH could reduce platelet aggregation induced by thrombin and inhibit platelet aggregation in response to adenosine diphosphate (ADP) [35]. In the present study, compared to Splenectomy group II, both the incidence of PVST and platelet aggregation were significantly decreased in LMWH group (*p* < 0.05 and *p* < 0.001, resp.). However, two rats in LMWH group died before euthanasia due to intra-abdominal hemorrhage, suggesting that the anticoagulants under such conditions should be used more carefully. We speculated that a possible reason might be the increased release of tissue plasminogen activator and consequent fibrinolysis induced by LMWH.

One of the most studied actions of RES was its antiplatelet aggregation property. RES exerted this function through multiple mechanisms. First, RES could inhibit both type I collagen mRNA expression and platelet adhesion to the collagen, which was the initiation of platelet activation [36]; second, RES could interfere with platelet aggregation through inhibiting Ca^2+^ influx into thrombin-stimulated platelets [37]; third, RES could reduce tissue factor (TF) activity by inhibiting nuclear factor-*κ*B/Rel-dependent transcription in both endothelial cells and monocytes [38].

Besides, RES was shown to reduce oxidative stress by different mechanisms including activation of antioxidant enzymes, inhibition of nicotinamide adenine dinucleotide phosphate (NADPH) oxidases, and chelation of metal catalysts [39], thus reducing the formation of ROS. Moreover, RES could enhance NO production by stimulated platelets via promoting the phosphorylation of protein kinase B and vasodilator stimulated phosphoprotein (VASP) [39]. RES also increased NO production by increasing NO synthase activity (NOS) [40]. All these activities also contributed to the antiplatelet aggregation activity of RES.

Of late, there were reports of plant-derived molecules, such as RES, causing platelet apoptosis [41, 42]. Further studies showed that RES triggered platelet apoptosis by multiple mechanisms including caspase-9, caspase-3, and caspase-8, gelsolin and actin cleavage, truncated Bid (tBid) formation, Bcl-2-associated X protein (Bax) translocation, cytochrome c release, phosphatidylserine (PS) exposure, and dissipation of the mitochondrial membrane potential, suggesting that RES-induced platelet apoptosis may be mediated by both intrinsic and extrinsic apoptotic pathway [43].

A previous study had reported that RES exerted protective effect on rats subjected to PVST via its antioxidant and antiaggregant properties [21]. The authors found that, compared with the rats subjected to PVST alone, significant increases in both tissue and plasma levels of reduced glutathione (GSH), as well as tissue c-AMP level, and decreased tissue malondialdehyde (MDA) level were observed among rats receiving RES (60 mg/d per nasogastric tube) for 10 days before being subjected to PVST. However, the effect of RES on the formation of PVST after splenectomy remains to be elucidated.

In the present study, we investigate the preventive effect of RES on the formation of PVST after splenectomy in a rat fibrosis model. The reported interval between splenectomy and PVST development varied and in our previous study the interval was 4 days [23]. Taking this into consideration, we choose 1 week as the end point of observation. Our results showed that the incidence of PVST in RES group, similar to LMWH group, was significantly decreased compared to Splenectomy group II, and no animal in RES group died due to postoperative complications such as bleeding. Moreover, we further studied the effect of RES on platelet aggregation, platelet ROS production, platelet NO production, and platelet apoptosis. As expected, platelet aggregation and platelet ROS production were significantly higher in Splenectomy II group rats than those in rats without liver cirrhosis 1 week after operation, while pretreatment with RES (50 mg/d per nasogastric tube for 10 days) showed a significant decrease in platelet aggregation and platelet ROS production than in Splenectomy group II. Besides, our results demonstrated that platelet NO production was significantly decreased in Splenectomy group II rats than that in rats without liver cirrhosis 1 week after operation, while pretreatment with RES showed a significant increase in platelet NO production than in Splenectomy group II. These findings affirmed the results of previous studies showing that ROS act as second messengers to activate platelets via NO inactivation [44]. PS exposure on cellular surface represents the most universal and best characterized target recognition signal, leading to phagocytes-mediated clearance of the apoptotic cells [45]. Our results also demonstrated that, in RES group, statistically significant increase in Annexin-V positive platelets representing PS exposure was observed when compared with the other four groups. More importantly, we provided the first evidence supporting the preventive effect of RES on the formation of PVST after splenectomy in the setting of cirrhotic, which are consistent with previous findings, suggesting the antiplatelet aggregation, antioxidative, vasorelaxing, and apoptosis-promoting effects of RES [39, 41–43]. However, additional studies with longer follow-up time are needed to affirm the results.

Taken together, our present study has provided evidence for the first time that RES exerts its preventive effect on PVST formation after splenectomy, through simultaneously inhibiting aggregation, decreasing ROS, and increasing NO production, as well as stimulating apoptosis effects on platelets, in a rat fibrosis model. RES, as a natural substance found in multiple plants, could be a promising candidate in the development of an effective antithrombotic therapy for the prevention of PVST after splenectomy for cirrhotic patients.
